# Prevalence of HBV and HIV coinfection from an infectious disease hospital in Mumbai

**DOI:** 10.1186/1471-2334-14-S3-P11

**Published:** 2014-05-27

**Authors:** Jayanthi Shastri, Nita Gangurde, Manish Pathak, Sandhya Sawant, Sachee Agrawal

**Affiliations:** 1 Department of Microbiology, TN Medical College and BYL Nair Charitable Hospital, Mumbai, India

## Background

Hepatitis B virus (HBV) and HIV coinfection are pressing health problems in developing countries including India. Concurrent infection with HBV may alter the natural history and treatment of both diseases. Hepatitis B and HIV frequently coexist due to common modes of transmission. HIV and HBV coinfection leads to complex immunopathological disorder heralding poor prognosis and dismal outcomes. The present study was aimed to examine the prevalence of HIV and HBV co- infection among hospitalized patients.

## Methods

Two groups of population were studied; group I (n=1000) hospitalized adult patients with clinical jaundice were tested for HBsAg by ELISA. HBsAg positive patients were further tested for HIV infection as per NACO guidelines. Group II (n= 650) hospitalized patients with symptoms like fever > 1month, weight loss >10%, cough >3 weeks and lymphadenopathy were tested for HIV as per NACO guidelines. HIV positive patients were further tested for HBsAg by ELISA.

## Results

In group I, 10 % were positive for HBsAg and out of 100 HBsAg positive 6% were co infected with HIV. In group II, 15.38% were positive for HIV and out of 100 HIV positive 25% were co- infected with HBV. Overall co infection is 15.5% with male preponderance and heterosexual mode of transmission being the commonest.

## Conclusion

HIV and HBV share common modes of transmission, therefore all HIV positive patients should be screened for HBV coinfection. Guidelines should be modified to include mandatory HBV testing among HIV positive individuals to monitor progress of infection and improve treatment outcomes.

